# Address of Prof. Allen, Inaugurating Lecture Course of 1872–3, in Rush Med. College

**Published:** 1872-11

**Authors:** 


### Editorial.

In compliance with the expressed desire of the Faculty and students of Rush Medical College, we take the liberty of disregarding the wishes and doing violence to the modesty of our worthy senior, in publishing in full, without his approval, his introductory address, inaugurating the Course of Lectures of 1872–3.

Although the address was written most hurriedly, in the midst of unusually pressing professional engagements, it nevertheless commends itself to our judgment, as peculiarly appropriate to the occasion and circumstances under which it was delivered.

We believe that the Alumni of the College hail with joy the knowledge that their alma mater still lives, and feel sure that the medical profession at large will take pride in the energy and perseverance which have been manifested by its Faculty. The disasters which overwhelmed the College, and its triumph over them, are a part of the history of the medical profession, and in that connection every incident connected therewith deserves to be recorded. H.

ADDRESS BY PROF. ALLEN.

*Opinionum commenta delet dies*—or, by sufficiently free translation, We change our notions as we get older.

A man born blind, whose cataracts at adult age Brother Holmes removes, sees all things on a plane, like a Japanese picture, without perspective. It takes education to make him see curves and corners. A baby, taking its first lessons in walking, tries to move both legs at once, and from its contused head gains an inkling of the first law of progress—do one thing at a time, "Left foot first, mark time, forward march!" The children of a larger growth have to obey the same law. Except one first become as a little child he can in nowise enter the kingdom of knowledge.

Some old philosopher said: "The profoundest object of philosophy is to restore mankind to common sense." The superficial object is to weave all knowledge into a plausible system of opinion, a coherent web of doctrine—much too often, in fact usually, of spidery strength, Joseph-coated colors, and Penelope-woven duration. Some book-writing doctors occur to my mind in this connection, many dead and some living; but, as suitable in a well-constructed sermon, the application is to be left to the latter part of the discourse.

The object of education and study, largely, is to correct the wrong impressions which our senses convey. Some people think that everything of mind comes from the senses. Books have been written to defend this notion—just as other books are written to prove, or at least convince, that man, "noble in reason, infinite in faculties," has developed through oysters, frogs and monkeys, from the original monad or tremulous protoplasm. Something more than good eyes is necessary to see, and what we hear is by no manner of means always truth. The world seems to stand still, and the sun and stars, fixed in a blue or black hollow sphere, to rise and set. The earth looks flat wherever one travels. The baby reaches after the moon, and many adult people believe they can "pluck out the heart of the mystery" of their own existence, the whence and wherefore of the universe, by similar exercise of the "appetencies." Savans in abundance will tell you exactly how many thousand years it has taken for Niagara Falls to wear its way back from the Ontario to the present precipice. They will figure out mathematically how long it took the coal strata to be deposited, and the coral reefs to be built.

Some doctors will tell you exactly how life sprang into being from deft, yet fortuitous, combination of atoms, and how all the varied phenomena of disease and remedy may be caught and imprisoned in the confines of a rhetorical sentence. They have done it for ages, and they are doing it now.

Did it ever occur to you that the confusion of tongues which fell upon the race at Babel, like many another so-called punishment, has proved, to our profession particularly, a wonderful blessing? Heaven in its goodness conceals from us in the unknown tongues many a vagary, wild dream or foolish imagining that otherwise we should have added to the already enormous catalogue of follies of opinion in our own vernacular. "Great is silence," and great is the *unknown* tongue!

In the macrocosm of history, opinions, doctrines, dogmas and notions appear and disappear like the shades and colors of the kaleidoscope as it passes from hand to hand, or even a muscle vibrates. In the microcosm of the individual life the dance of the atoms proves equally perplexing, and the mental phantasmagoria almost to the same degree illusive. I say almost, for, as Gibbon writes, "The experience of past faults which may sometimes correct the mature age of an individual, is seldom profitable to successive generations of mankind." Experience is the report of the senses, corrected by the judgment, or highest power of the intellect. And this is exactly where many people mistake. "To most people," as Coleridge writes, "experience is like the stern lights of a ship, which illumine only the track it has passed." There is an old fable of the time when the animals were gifted with language (before their Babel was). "An asse and a mule went laden over a brook; the one with salt, the other with wool; the mule's pack was wet by chance, the salt melted, his burden the lighter, and he thereby much eased; he told the asse, who, thinking to speed as well, wet his pack likewise at the next water, but it was much the heavier; he quite tired."

A weighty experience for the poor ass, but practically exemplified every day among bipeds who yet talk.

So far as history gives us any clue, men have been very much the same through all the ages. Surroundings have varied immensely; exterior influences have been continuously changing, but the human mind has ever manifested the same workings. There is probably not a person here present but who, within his own memory and observation, has seen the most remarkable changes in public opinion and belief—political, moral, scientific, and social. Reading the history of the world intelligently teaches us not to be astonished at this. "The individual is an enigma, the mass a mathematical problem." As we recede from objects too vast or too multitudinous for us to comprehend, they concentrate to dimensions and shapes within the limit of our faculties. As we approach what seems both single and simple, we find it resolving into numerous and complex parts. In our study of medicine, or indeed any branch of science, it becomes necessary to use both telescopic and microscopic vision—literally and by striking metaphor.

A few moments since, I said the savans, some of them, will tell how long things have been making or shaping. They will figure out to you, with astounding nearness, about how long it has taken to develop a crystal or a polyp to a Shakespeare or an Agassiz. On the basis of what they call experience, personal or .historic, they will give you the "testimony of the rocks," and from the fossils of the lowest strata will evoke confirming voices.

How trivial and small all this is to the real thinker, who can look above and beyond the "muck-rate." As the senses are the servitors of the intellect, so experimenters and observers are the hewers of wood and drawers of water to the real philosopher.

Every school-boy has been taught that the earth revolves upon its axis—hence darkness and daylight; that the moon revolves around the earth—hence poets' rhapsodies and lovers' walks by moonlight, and garroters and policemen at other hours; that the earth revolves about the sun—hence spring, summer, autumn, and winter. But a still larger and more magnificent view, which centuries of history give us only in minutest, yet most readable, characters, exhibits to us the colossal fact that our sun and its attendant train of planets do not swing forever in the same space relatively to the so-called fixed stars. Our solar system is steadily moving in one particular direction, from which it results by optical law that the stars which it is receding from appear to be approaching each other, and those which it is approaching are gradually separating more and more. From the analogy of the motion of all the sidereal bodies, this motion is most likely in an ellipse of revolution. As the earth with its satellite moon revolves around the sun, so does our solar system revolve in company around some inconceivably far-away luminary or central sphere. And that grand orbit gives a spring, a summer, autumn, and winter, each of myriad thousands of years. In that spring the amazing flora and fauna of the geological strata were warmed into being; they matured in that summer of summers; they declined in that wonderful autumn of thousands of years; and the inevitable winter of the ages buried them in the solid rocks or ice which covered the globe —in our time to be dug up and maundered over, and development theories fashioned from them. "O lame and impotent conclusion!"

Experience, to be of advantage, even to the individual, necessitates the possession of sound judgment and wide acquaintance with facts; in other words, general knowledge. No man can thoroughly understand the branch of science to which he especially devotes his attention, unless he ascends first to the level of all sciences. In medicine, for instance, we exclaim against the so-termed specialties, unless the specialist has first gone over the entire curriculum of medical study. He must have risen to a level where he could see the relations of things, as well as the things themselves. In pictures of battles the commanding general is usually represented in front of his troops on a showy Bucephalus, rich in epaulettes and buttons, waving a huge sword over his head, looking like an incarnate Mars. The fact is, I am told, the general is behind all the ranks, up a hill, or a tree, or a house-top, very properly careful of his personal carcass, and yet seeing to the relations of things. But before getting to be a general, a man should know the manual of arms, and all duties of privates and subordinate officers. The medical student who wishes to be able to command experience to his service, and to acquire a sound philosophy or system whereby he may be guided in the subsequent duties of his professional life, must first thoroughly master his manual of arms, tactics, fortifications, *et id omne genus.* Time enough after that for his highest efforts in "grand strategy." Patrick Henry said: "The only lamp by which my feet are guided is the lamp of experience" —quite enough light, perhaps, for a politician or stump speaker; but if you expect this to answer your purpose hereafter, I am afraid your lamp will turn out a mere French candle—*bougie*, I think they call it—very tallowy, serving mainly to make darkness visible.

We change our notions as we get older; at least we ought to do so. There are some people—doctors even—who pride themselves upon what they call their consistency, that is, their obstinate adhesion to opinions and notions instilled into their minds in the far-away days of their pupilage. The sins of the fathers are visited upon the children, even unto the third and fourth generation. Thus the old-time experience is hung upon the shoulders of the young student, like Christian's pack in "Bunyan's Pilgrim's Progress."

Now this is all wrong. It is the inorganic world in which absence of change is admirable. But where life is, there is incessant change. Nay, without this change there is no manifestation of life. The wasting away of the old and the assumption of new forms is the essential fact of life. The old mythology represented the genius of Life as inverting his torch and becoming the Angel of

Death, but the philosophic physiologist will tell you that the truer allegory is that of the grand old Norse conception of the life-tree Igdrasil, which sends down its roots deep into the death regions. The *dead* dragon's teeth sowed sprung up in crops of armed men. "Truth advances only over ruins"—the ruins of previous beliefs. We, old men, are exceedingly apt to lament the degeneracy of these latter days, and in so doing prove the coming on of our days of dotage. The soil of mind is only then rendered truly fertile when the rank weeds of prejudice, false doctrine, crude judgment and foolish fancies are alike uprooted and plowed under. And it is best to do this as early as possible, else these tares will "go to seed," and by and by, when you undertake to become free from them, they will become more abundant by some twenty, some sixty, and some a hundred fold. Time comes when Ephraim is joined to his idols, and is to be let alone. Consistency is a jewel neither for a swine nor a mule's snout. In a presentable form it suggests that he who wears it has a sound and coherent philosophy of life and action, of principles and practice—not sequence in time only. The consistent mind is one wherein there is congruity among its several parts, and agreement with itself. The opinions it forms and the actions it incites stand together in an orderly manner, according to Heaven's first law. The consistent teacher, speaker or writer will not contradict in one part of his discourse the doctrine of another part. The consistent practitioner will not in one case adopt different principles of management from those which guided him in another. Adopting a principle which he thoroughly believes to be sound, no dictum of antiquity or usage of the present, will swerve him from carrying it into both teaching and every-day practice.

Like a sculptor, in whose mind glows the beautiful ideal, he is constantly separating from the marble blocks those obstructing masses or particles that preclude him from full view of the inimitable statue within. He has no regrets at the parting with these.

And thus the consistent, enthusiastic medical scholar zealously labors at the work before him, until at last, like Prometheus of olden myth, he calls down the *divinam particulam auris* to vivify his creation—and then, usually, gets from men the reward Prometheus got for his pains—a consignment of his liver to the vulture, so far as they care. On the whole, the best of us get so little ahead of the poorest that it is not well to "gush" extravagantly over the matter.

My young friends, although I would not be so presuming or indecorous as to charge any one of you with the cultivation of a crop of "wild oats," nevertheless I will venture to remark (confidentially) that the very clearest head among you needs, nay, absolutely demands, that minute knowledge and, at the same time, that comprehensiveness of thought which will enable it to weed out the prejudices, fancies and notions which will gain admission to the very best of brains. In the book of Job we read that when the sons of God were gathered together, Satan came also, and just so it happens that no man living is exempt from some one meddlesome demon. Happy if the name be not "Legion." He who recognizes his attendant Sathanas is not happy, but tolerably safe. He who confidently believes he is not possessed of such a devil may be triumphant and self-satisfied, but Mephistopheles grins over his shoulder, and some day he will disappear in a fire-wagon quite other than that the prophet saw in the vision.

Some of you have a fair stock of elemental medical knowledge already on hand. Do not be afraid of adding to it. When Xerxes looked over his great army, they say he wept because in a hundred years he and they would all be dead, and so I have myself, on occasions like the present, shed metaphorical tears at thought that, in ten years even, many of the class would not know nearly so much as now.

In the old countries the incipient Esculapius enters, like an incipient carpenter, upon a regular apprenticeship, the first years of which are notably devoted to the acquisition of a smattering of academical studies, reading, writing, arithmetic, history, "a little Latin and less Greek;" sweeping out the office, running of errands; by and bye advanced to the position of a seat in his master's gig, he passes on to the hospital and lecture-room, and ultimately is raised to a full-fledged doctorate. In this country no such menial position is assigned the student. He usually has already acquired a reasonable general education, before he makes choice of a profession. Then at mature age, or verging upon manhood, he enters the office of some physician of repute in his immediate neighborhood. (And here, permit me to interpolate, let no man sneer at the country physician of good local repute. It is ten-fold easier for a man to conceal his real ignorance, acquire extensive practice, and even reputation, in a large city, than in a goodly-sized country town, where everybody knows everybody and weighs everybody.)

The American medical student, once started in the pursuit of his profession, will overtake and possess it. Self-reliant and determined, he becomes zealous and enthusiastic, as all native Americans are born, and adopted citizens become, in whatever they undertake. He will achieve success because he knows its essentials are under the command of his own will. In his vocabulary there is no such word as fail. He rejoices as a strong man to run a race. The maxim is, "What man has done man may do." If my predecessors have attended six lectures, an hour each, per day, I can do it. Away with the puny, pitiful whining about too much to do. It is unworthy the age—unworthy, especially, the American medical student. One thing at a time, but to that one thing is fastened everything of energy, everything of faculty. No cork-floats or bladders for the one who wills to swim. You are here face to face with facts; you have no windmills of dogma to attack, nor are you called upon to incise the wine-skins of old-time theory.

Explanations are temporary, fleeting and changeable, but facts are ever silver, gold and diamonds, whatever their temporary impress or setting. Get them now, and in all the abundance possible; quarrel about them hereafter. You have not time for dissensions or debate now. You will get wiser as you grow older, or, at least, change your notions. Sufficient unto the day is the evil thereof. Let the "eclectic" rage, and the homœopathist imagine a vain thing; you are employed in your own great work, and like him who rebuilt the temple, you cannot come down.

Of the making of books there is no end, especially of medical books without beginning or end. Do not be frightened. You are not obliged to read them. In those countless bushels of chaff there are many grains of wheat. We, your temporary preceptors, have been long engaged in looking over the heap and selecting kernels for your particular nourishment and comfort. (O, what cackling have we heard over a single grain in times not long past!) The winds of time, to our encouragement and satisfaction be it said, are constantly wasting the heap; there are hosts of errors and follies that will blow away, without assistance from our respiratory muscles.

You need not trouble yourselves about divisions among medical men, different schools of teaching or practice. They that cast out the devils of ignorance and folly are with us and for us. Time will dissipate the largest cloud of wrong doctrine, and, given plenty of rope, the speculators and theorists will hang themselves.

Somebody remarked: "The world moves!" We cordially assent to the statement. Rush Medical College moves also. A year ago, some of you will recollect, it was located on what is everywhere known as the "North Side."

The geologists tell us that what are now the polar latitudes were once habitable and inhabited, but owing to causes over which the natives had no control, the climate became so inclement that ultimately it ceased to be desirable, physiologically, for business or residence purposes. *Mutatis mutandis* — this was the fact about a year ago on the North Side. Hence Rush Medical College migrated, and has gone into camp or winter quarters, where we are now present. Some one has said Rush Medical College was burned down last year, during the general house-warming in which our city indulged. There can be no greater mistake. I have high authority for the remark that "something more than bricks and mortar is necessary to make a medical college." For many years Rush College has been demonstrating the truth of the proposition. Our splendid and commodious building went up in flame and down in ashes, but the College only moved to the South Side, preparatory to even yet another migration. "Out of the nettle danger it has plucked the flower safety." Scarcely was the college building completed before we began to recognize the inconvenience of being at such a distance from the great hospital of the city. We hoped, it is true, for the establishment of a respectable hospital on the North Side, but circumstances seem to have combined to postpone for too long a time this desirable event.

The fire came and solved the problem. As the mountain would not come to Mahomet, Mahomet went to the mountain. Rush Medical College moved.

Without the preliminary and accompanying collegiate instruction and discipline hospital experience is little worth. Without hospital illustration, the college plays Hamlet with the part of Hamlet omitted with malice aforethought. The college and the hospital should be side by side, and the clinical teacher and didactic instructor move hand in hand. What to observe and how to observe, and then what is the meaning of the indications? These are the great questions, and the student who faithfully seeks their solution will succeed. Power gained to solve these will bestow power to solve others, however recondite, with regard to right methods of cure.

One thing at a time. Do not be in a hurry to get at what you fancy the practical portion, before you have mastered the often-wearisome elementary parts of the matter. Before you make chicken soup, catch and kill your chickens.

The other day, when I was notified that the annual greeting to the new class was to be pronounced by myself, it must be confessed, I was more than half disposed to shrink from what has always heretofore been a pleasure. The surroundings are strange. For many years the old associations have clustered about the corner of Indiana and Dearborn streets. It seems something like going from the old home, abandoning the family roof tree. I half feared I should feel as though we had all left Rush and gone to some new-fangled reform school.

Then, again, it was reasonable to expect that the speaker of the evening should be immense and flamboyant over the great blaze; that his sentences should corruscate and glow like the lambent flames which swept away more of the works of men than, in the world's history, ever disappeared in any single holocaust.

But when it was remembered that so many of those who have stood before successive classes of Rush Medical College for many years, were still responsive to the roll-call for their usual duty, there could arise no other emotion than a stronger determination than ever that the pillars of this temple shall never be shaken, or the fires upon its altars ever go out. And then it occurred, What is the use talking about the fire? Can we "gild refined gold, paint the lily, or add colors to the violet?" Of course we may—but lose the labor. But the tongue wags not now, nor is the pen yet shaped from steel, gold, or goosequill, which may describe the Chicago fire. None but itself can be its parallel.

One old notion, fortunately, was burned up at the same time with old Chicago—that long years were necessary in which to build a great city. In these times if a thing needs to be done it is done, and done quickly.

In this study of ours, medicine, a similar idea stands pre-eminent. You can build your professional temple *cito, tuto et jucunde* if you imitate in zeal and industry the builders of new Chicago. With these in abundance on your part, and competent instructors, such as I am proud and happy to rank my colleagues, you can attain in the prescribed curriculum necessary to be passed over before examination for your diplomas, not only the degree, but positive knowledge and experience sufficient to start you in professional life, at once, as safe and successful practitioners.

Without these, no matter how elongated your term of pupilage, how complex the plan of education, and how plausible the teacher, the finally won diploma is but a frail raft in a stormy sea. Rush Medical College points proudly to its alumni, wheresoever dispersed, in absolute proof of these propositions. By their success in every department of the profession it is willing, in the future as in the past, to be tried and tested.

We meet you to-night not in a temple of science, stately with towers, groined arches and clustering columns. You look about you and see none of these. The ancient Spartan, when the astonished visitor asked for the fortifications of the city, pointed to the *men* and said: "These are the walls of Sparta." We welcome you to a similar enclosure, and pledge the highest efforts of the Faculty for your rapid and sure advancement.

